# Methyl Selenol as a Precursor in Selenite Reduction to Se/S Species by Methane-Oxidizing Bacteria

**DOI:** 10.1128/AEM.01379-19

**Published:** 2019-10-30

**Authors:** Abdurrahman S. Eswayah, Nicole Hondow, Andreas C. Scheinost, Mohamed Merroun, Maria Romero-González, Thomas J. Smith, Philip H. E. Gardiner

**Affiliations:** a Biomolecular Sciences Research Centre, Sheffield Hallam University, Sheffield, United Kingdom; b Biotechnology Research Centre, Tripoli, Libya; c School of Chemical and Process Engineering, University of Leeds, Leeds, United Kingdom; d The Rossendorf Beamline at ESRF, Grenoble, France; e Institute of Resource Ecology, Helmholtz Zentrum Dresden Rossendorf, Dresden, Germany; f Department of Microbiology, University of Granada, Granada, Spain; g School of Engineering and Materials Science (SEMS), Queen Mary University of London, London, United Kingdom; North Carolina State University

**Keywords:** selenite reduction, elemental selenium, methane-oxidizing bacteria, mixed chalcogenide amorphous nanoparticles

## Abstract

A wide range of microorganisms have been shown to transform selenium-containing oxyanions to reduced forms of the element, particularly selenium-containing nanoparticles. Such reactions are promising for the detoxification of environmental contamination and the production of valuable selenium-containing products, such as nanoparticles for application in biotechnology. It has previously been shown that aerobic methane-oxidizing bacteria, including Methylococcus capsulatus (Bath), are able to perform the methane-driven conversion of selenite (SeO_3_^2−^) to selenium-containing nanoparticles and methylated selenium species. Here, the biotransformation of selenite by Mc. capsulatus (Bath) has been studied in detail via a range of imaging, chromatographic, and spectroscopic techniques. The results indicate that the nanoparticles are produced extracellularly and have a composition distinct from that of nanoparticles previously observed from other organisms. The spectroscopic data from the methanotroph-derived nanoparticles are best accounted for by a bulk structure composed primarily of octameric rings in the form Se_8 −_ *_x_*S*_x_* with an outer coat of cell-derived biomacromolecules. Among a range of volatile methylated selenium and selenium-sulfur species detected, methyl selenol (CH_3_SeH) was found only when selenite was the starting material, although selenium nanoparticles (both biogenic and chemically produced) could be transformed into other methylated selenium species. This result is consistent with methyl selenol being an intermediate in the methanotroph-mediated biotransformation of selenium to all the methylated and particulate products observed.

**IMPORTANCE** Aerobic methane-oxidizing bacteria are ubiquitous in the environment. Two well-characterized strains, Mc. capsulatus (Bath) and Methylosinus trichosporium OB3b, representing gamma- and alphaproteobacterial methanotrophs, respectively, can convert selenite, an environmental pollutant, to volatile selenium compounds and selenium-containing particulates. Both conversions can be harnessed for the bioremediation of selenium pollution using biological or fossil methane as the feedstock, and these organisms could be used to produce selenium-containing particles for food and biotechnological applications. Using an extensive suite of techniques, we identified precursors of selenium nanoparticle formation and also found that these nanoparticles are made up of eight-membered mixed selenium and sulfur rings.

## Abstract (precis)

Aerobic methane-oxidizing bacteria are ubiquitous in the environment. Two well-characterized strains, Mc. capsulatus (Bath) and Methylosinus trichosporium OB3b, representing gamma- and alphaproteobacterial methanotrophs, respectively, can convert selenite, an environmental pollutant, to volatile selenium compounds and selenium-containing particulates. Both conversions can be harnessed for the bioremediation of selenium pollution using biological or fossil methane as the feedstock, and these organisms could be used to produce selenium-containing particles for food and biotechnological applications. Using an extensive suite of techniques, we identified precursors of selenium nanoparticle formation and also found that these nanoparticles are made up of eight-membered mixed selenium and sulfur rings.

## INTRODUCTION

A key biotransformation mechanism of most microorganisms exposed to selenium oxyanions is dissimilatory reduction to nanoparticulate elemental selenium (1–4). The formation of the nanoparticles (NPs) reduces the toxicity and the bioavailability of the selenium species (5–9). Not only does the formation of the NPs reduce the adverse environmental impact of the oxyanions on the microorganisms and their surroundings but also it presents an approach for the production of selenium NPs (SeNPs) designed for a variety of technological, clinical, analytical, and industrial applications (10–20). There is a demand for nanoparticles with new combinations of properties for specific applications. In the case of selenium-containing particles, to date these have included exploitation of their optical properties to improve the response of sensors (11), binding of heavy-metal ions to effect remediation of pollution (12), and exploitation of the antitumor (16), immunomodulatory (17), and antimicrobial (19) activities of specific types of selenium-containing particles.

Different bacteria are known to convert selenium oxyanions into red amorphous SeNPs with distinct features. Selenium NPs produced by the selenium-respiring bacteria Sulfurospirillum barnesii and Bacillus selenitireducens contain predominantly Se_6_ chain units, while Selenihalanaerobacter shriftii has Se_8_ rings (21). Desulfovibrio desulfuricans, a sulfate-reducing bacterium, produces amorphous spherical submicron particles containing selenium and sulfur (22). Selenium NPs produced from selenite by Azospirillum brasilense are composed of cyclic Se_8 − *x*_S*_x_*, with Se_6_S_2_ being the most likely structure (8). Azospirillum thiophilum produces amorphous selenium particles with no evidence of either Se—S or S—S bands in the Raman spectrum (23). Stenotrophomonas bentonitica produces amorphous Se^0^ nanospheres that subsequently transform to a one-dimensional (1D) trigonal selenium nanostructure where sulfur is associated with the SeNPs (24). In an earlier study (25), we presented indirect evidence from transmission electron microscopy (TEM) imaging with energy-dispersive X-ray spectrometry (EDX) measurements to show that sulfur is associated with amorphous selenium in the extracellular NPs that are produced in a methane-dependent fashion from the reduction of selenite by the aerobic obligate methane-oxidizing bacteria Methylococcus capsulatus (Bath) and Methylosinus trichosporium OB3b.

Besides the above-mentioned studies on the biotransformation of selenium oxyanions, most investigations have focused on the formation of the NPs. A few others have identified the concomitant release of volatile selenium species into the headspace gas (26, 27). However, none of these approaches has provided enough information to enable the elucidation of the processes leading to the formation of the amorphous NPs. Indeed, the formation of the extracellular amorphous NP forms, as reported in many studies (18, 21, 28–30), may indicate a limited direct involvement of the microorganisms in their formation, although the initial reactants may have been produced in the bacteria. The size of the extracellular nanoparticles produced by Mc. capsulatus Bath increased with time (25), which suggested that the growth of the NPs is a result of abiotic reactions in the culture medium outside the cells. The formation of mixed chalcogenide species by exchange reactions, when both Se and S species are present in the gaseous and solution phase, has been reported (31, 32). It is probable that similar reactions occur in the culture medium solution, eventually resulting to the formation of the nanoparticles. Here, it was our intention to observe the process of nanoparticle formation and the structure of the nanoparticles in greater detail and to be better able to understand the biotic and abiotic transformations occurring in the methanotroph-mediated transformation of selenite. To this end, both selenium- and sulfur-containing species were sampled from the headspace and solution of selenite-amended and control samples at fixed times by sorptive extraction in conjunction with analysis by thermal desorption (TD)-gas chromatography (GC)-mass spectrometry (MS) to identify the compounds. In parallel, the formed NPs were characterized by a range of physical techniques, namely, attenuated total reflectance Fourier transformation infrared spectroscopy (ATR-FTIR), Raman spectroscopy, transmission electron microscopy (TEM) and energy dispersive X-ray (EDX) spectrometry, X-ray absorption spectroscopy (XAS), and X-ray photoelectron spectroscopy (XPS). Herein, the results obtained from these measurements are used to inform the formation and elucidation of the structure of the sulfur-doped red amorphous selenium NPs produced when a methane-oxidizing bacterium reduces selenite. Mc. capsulatus (Bath) was chosen for these experiments since it transforms selenite at approximately five times the rate of Ms. trichosporium OB3b (25).

## RESULTS

**FIG 1:**
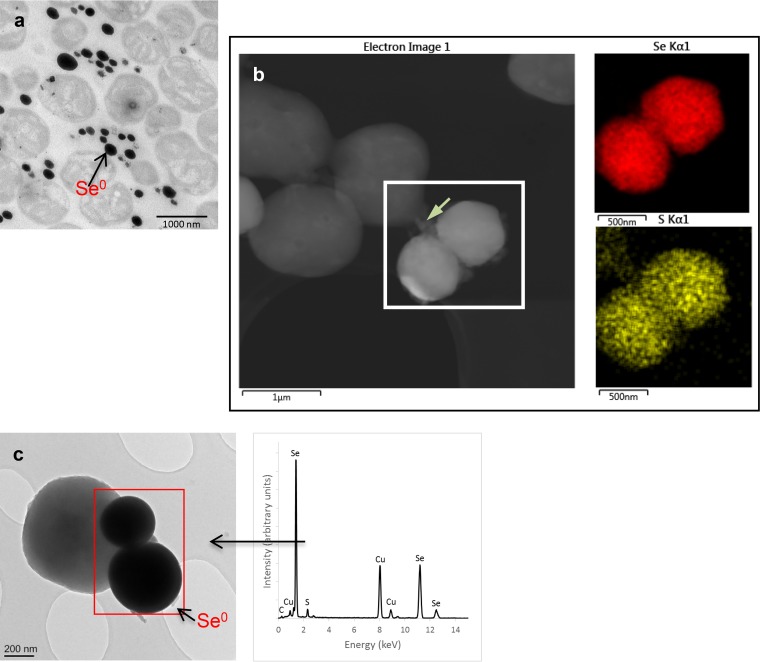
TEM thin-section micrographs of Mc. capsulatus (a) exposed to 20 mg liter^−1^ SeO_3_^2−^, showing the extracellular locations of the Se^0^ nanospheres; HAADF-STEM imaging, showing Se nanospheres associated with the cells with EDX maps (generated from spectra collected from the indicated areas) of Se and S (b); and TEM of Mc. capsulatus cultures exposed to 20 mg liter^−1^ SeO_3_^2−^ (c) with EDX analysis within the electron-dense regions (Se^0^ nanospheres). The green arrow in panel b indicates the trail of sulfur-containing material between the cells and the particles, which may indicate partly particle-associated proteinaceous material derived from the cells. Cells were fixed with 3% glutaraldehyde and 2% OsO_4_ immediately before the analysis.

Previous investigations with the methanotroph species Mc. capsulatus (Bath) showed that the sizes of the NPs grew rapidly from an average of 220 ± 51 nm in the first 4 h to about 400 ± 77 nm in the next 44 h (25). Here, high-angle annular dark-field scanning transmission electron microscopy (HAADF-STEM) imaging was performed and TEM thin-section micrographs of cultures producing the nanoparticles were obtained, and it appears that the NPs are associated extracellularly with the cells (Fig. 1a). Furthermore, the spatial distributions of Se and S in the EDX maps overlap, indicative of a spatial and likely structural association, suggesting the formation of mixed chalcogenide nanoparticles (Fig. 1b). The intensity of the Se signals was, however, much higher than that for S (Fig. 1c). In addition, examination of the S_kα_ map (Fig. 1b) revealed that not only was there sulfur in the particles but also there was a trail of the element linking the particles to the cells, suggesting likely sulfur-containing proteins from the bacterial cells.

The Se K-edge X-ray absorption near-edge structure (XANES) spectra of the particles formed by Mc. capsulatus (not shown) were similar to those of red amorphous Se, with no detectable residual selenite being present in the samples. Previous shell fitting of the extended X-ray absorption fine-structure (EXAFS) spectra formed by Mc. capsulatus (25) showed the characteristics of amorphous elemental Se^0^, with two atoms at a distance of 2.35 Å; however, this is slightly shorter than the 2.36 Å expected for red elemental Se (33, 34). The shorter bond length is an indication that there may be a partial substitution of Se for the smaller S atom in the structure of the amorphous red elemental Se. This is a feature that has been observed for mixed Se and S nanoparticles formed by A. brasilense (8). In order to unravel the makeup of the nanoparticles, the surfaces of the particles were characterized by FTIR, XPS, and Raman spectroscopy.

### FTIR analysis.

**FIG 2:**
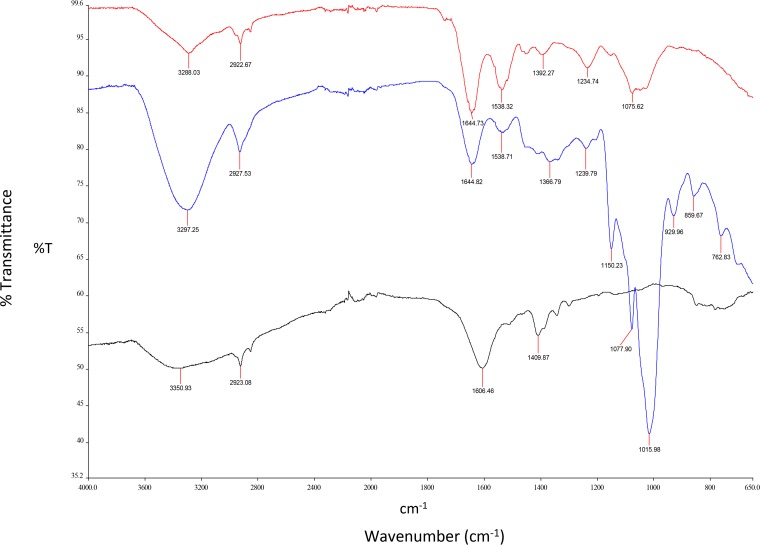
FTIR spectra of freeze-dried Bio-SeNPs (blue) and bacterial biomass (red) of Mc. capsulatus, exposed to 20-mg liter^−1^ SeO_3_^2−^ and harvested at an OD_600_ of ∼0.7, separated by centrifugation, washed with phosphate-buffered saline, pH 7.2, and freeze-dried, as well as those of the Chem-SeNPs (black), obtained through reaction of Na_2_SeO_3_ with l-cysteine. The spectra are representatives of those from 5 runs of the experiments.

The FTIR spectra of the freeze-dried selenium nanoparticles produced by Mc. capsulatus in liquid nitrate mineral salts (NMS) medium amended with selenite (Bio-SeNPs), the chemically synthesized SeNPs (Chem-SeNPs; the in-house-produced selenium nanoparticles described in Materials and Methods), and the bacterial biomass are shown in Fig. 2. The assignments of the bands, which are based upon the characteristic vibrational frequencies of the different types of covalently bonded atoms, are summarized in Table 1. The peak centered at 3,297 cm^−1^ corresponds to the —OH and —NH stretching vibrations of the amine and carboxylic groups. Peaks at 2,927 cm^−1^ correspond to the aliphatic saturated C—H stretching modes (30, 35). The peaks at 1,644, 1,538, and 1,239 cm^−1^ are characteristic of amide I, amide II, and amide III bands of proteins, respectively (36, 37). The symmetrical stretch of the carboxylate group can be attributed to the bands observed at 1,366 cm^−1^. The peaks at 1,150, 1,077, and 1,015 cm^−1^ correspond to the C—O stretching vibrations of C—O—C groups (36, 38). The presence of phosphoryl groups was confirmed by the peak at 929 cm^−1^. Additionally, the peaks at 859 and 762 cm^−1^ (fingerprint region) could be mainly attributed to the aromatic ring vibrations of aromatic amino acids (tyrosine, tryptophan, phenylalanine) and, possibly, nucleotides (39, 40).

**TABLE 1:** Tentative assignments of main bands to the relevant functional groups^*a*^

Sample	Wave no. (cm^−1^)												
	O—H, N—H (amide A in proteins)	C—H (in >CH_2_)	C=O (ester moiety)	Amide I (in proteins)	Carboxyl (COO^−^)	Amide II (in proteins)	-CH_2_/-CH_3_ (in proteins, lipids, polyesters, etc.)	C=O of COO^−^	C—O—C/C—C—O (in ester moieties)	Amide III / O—P=O	C—O, C—C, C—H, C—O—C (in polysaccharides and polyesters)	Phosphoryl groups	Fingerprint region
Cell biomass of Mc. capsulatus	3,288	2,922		1,644		1,538		1,392		1,234	1,075		
SeNPs produced by Mc. capsulatus	3,297	2,927		1,644		1,538		1,366		1,239	1,150, 1,077, 1,015	919	859, 762
Chem-SeNPs		2,923			1,606			1,409					

a The data have been published previously (30, 35–40).

The FTIR spectra of the SeNPs of Mc. capsulatus differ from those of the bacterial biomass (control) and the Chem-SeNPs. The main difference between the spectra is that the Bio-SeNPs exhibited more peaks in the protein and polysaccharide vibration region, indicating the presence of proteins and polysaccharides in the biomacromolecules capping the SeNPs (20, 30, 41, 42).

In contrast, Chem-SeNPs obtained through the reaction of Na_2_SeO_3_ with l-cysteine displayed a broad absorption band at about 3,350 cm^−1^ and an absorption band at 2,923 cm^−1^ that are assigned to O—H vibrations of the absorbed H_2_O and C—H vibration in the alkyl chain of l-Cys, respectively. The peak at 1,606 cm^−1^ can be mainly attributed to C=O vibrations. It is noteworthy that organic residues, such as carbohydrates, lipids, and proteins, on the surface of biogenic SeNPs were completely absent in the Chem-SeNP spectrum (Fig. 2). The FTIR spectra of the Bio-SeNPs separated from the Mc. capsulatus cells showed bands typical of proteins, polysaccharides, and lipids associated with the particles.

### XPS analysis of the particle surface.

The surface composition of the harvested red particles was obtained by XPS, which is a surface technique that obtains information about the elemental composition of a sample according to the emission of photoelectrons when the sample is irradiated with X-ray energy. The signals observed in XPS can be identified, based on their binding energy, as arising from the expulsion of electrons from specific energy levels within each element on the surface (rather than the interior) of the particles. Hence, the surface elemental content can be summarized from the full-range XPS spectrum (see Fig. S1a in the supplemental material). In addition to the selenium, which is present at a concentration of 1.25% (atomic percent), there are five other elements, carbon (46.32%), oxygen (31.41%), nitrogen (8.61%), calcium (5.89%), and phosphorus (4.77%), that were detected on the surface of the particles (see Fig. S1a and the elemental information therein). The presence of the first three elements is an indication that there are organic molecules on the particle surfaces. The high-resolution spectral scans, which show the regions due, respectively, to Se, C, N, and O, and the assigned chemical species from the core level XPS spectra of C 1s, N 1s, and O 1s bands. are shown in Fig. S1b to e in the supplemental material. These spectra are consistent with the presence of biomolecules, including proteins, on the surface of the particles. The spectrum for Se 3d shows a doublet which does not show baseline resolution. However, the fitted deconvoluted peaks in this region (Fig. S1b) show two predominant bands at binding energies of 55.16 and 56.02 eV, respectively, and two minor peaks at 55.75 and 56.61 eV, respectively, with the latter pair of peaks resulting from the Se 3d peak split by spin orbit coupling into Se 3d5/2 and Se 3d3/2. The key conclusion enabled by these results is that the observed range of binding energies between 55.16 and 56.61 eV is indicative of the presence of reduced selenium species, including elemental selenium, at the surface of the particles (43). The binding energy expected for the Se 3d electrons in selenite (SeO_3_^2−^) is indicated in Fig. S1b; it is clear that no signal is observed at this position.

### Raman characterization of the amorphous particles.

**FIG 3:**
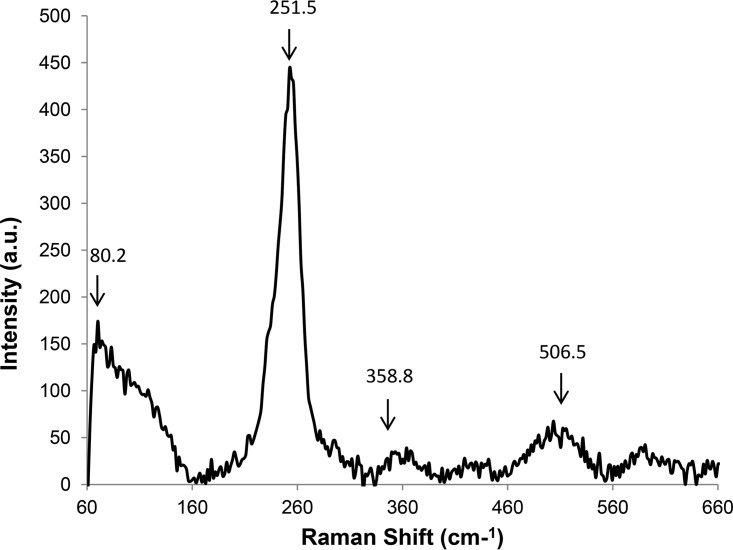
Raman spectra of purified Se nanospheres from Mc. capsulatus. a.u., arbitrary units.

Vibrational spectroscopy, particularly Raman spectroscopy (which is a visible light-scattering technique that gives information complementary to that from infrared [IR] spectroscopy) has been the technique of choice for the characterization of Se*_n_* allotropes and aggregates. The deconvoluted Raman spectrum obtained between 50 and 600 cm^−1^ showed a shift in the frequency of the scattered light that corresponds to the vibrational spectrum of the harvested SeNPs (Fig. 3). There are four bands which are visible: the main band at 251.5 cm^−1^ and smaller ones at 80.2, 358.8, and 506.5 cm^−1^. All of the bands were present in all of the scans of samples collected at different time points: 6 h, 24 h (Fig. S2), and 48 h (Fig. 3). The band at 506.5 cm^−1^ was more prominent than that at 358.8 cm^−1^. The band at 80.2 cm^−1^, which is a shoulder, was visible only in the deconvoluted spectrum.

It has been proposed that amorphous selenium is composed of a mixture of Se*_n_* rings and helicoidal chains (44–46). The proportion of each depends on the chemical and physical conditions under which the samples are made and the treatments to which they have been subjected. According to Carini et al., the band at about 250 cm^−1^ is characteristic of amorphous selenium (47). The symmetrical band at 251.5 cm^−1^, with a full-width half maximum of 30 cm^−1^ found in this study, is characteristic of Se-Se stretching vibration in pure Se_8_ (44, 48, 49), with its deformation vibration being at 80.2 cm^−1^. However, in the study of mixed selenium and sulfur alloys by Machado et al., the authors state that as the sulfur concentration increases in mixed amorphous selenium, the peak at 234 cm^−1^ usually associated with Se chains decreases in intensity and the band at 250 cm^−1^, usually associated with selenium rings, increases in intensity (50). They also observed the appearance of a band at 352 cm^−1^ as the selenium-to-sulfur ratio increases to either 4:1 or 7:3 (50). The band at 358.8 cm^−1^ has been assigned to S—Se stretching vibration (51, 52). The band at 506.5 cm^−1^ is probably due to the Se—Se overtone band of the fundamental band at 251.5 cm^−1^. The S—S vibration band is not seen when the S composition is below that found in Se_6_S_2_ (44). Therefore, it is probable that the composition of the harvested particles is Se_8 −_ *_x_*S*_x_*, where *x* is equal to or less than 2.

### Speciation of selenium and sulfur in the medium solution and headspace.

The GC-MS chromatograms of the species found in both the headspace and solution of the selenite-amended medium at 4 h and 20 h are shown in Fig. S3. The earlier time was chosen because the formation of the particles and, therefore, the red color of the solution were barely discernible. A summary of the selenium- and sulfur-containing species is given in Table 2. Examination of the data in Table 2 showed that after 4 h, three compounds, methyl selenol (CH_3_SeH), dimethyl selenenyl sulfide (DMSeS; CH_3_-Se-S-CH_3_), and dimethyl diselenide (DMDSe; CH_3_-Se-Se-CH_3_), were detected in both the solution and the headspace. In addition, dimethyl diselenenyl sulfide (CH_3_-Se_2_S-CH_3_) and bis(methylseleno) methane (CH_3_-Se-CH_2_-Se-CH_3_) were also found in the solution. At 20 h, dimethyl selenenyl disulfide (CH_3_-SeS_2_-CH_3_) was detected in the solution, in addition to triselenothone/dimethyltriselenide (CH_3_-Se-Se-Se-CH_3_), which was detected in both the solution and the headspace. The control in the absence of selenite showed no selenium-containing species, though three sulfur-containing species were observed (Table 2).

**TABLE 2:** Summary of the selenium- and sulfur-containing species detected in the headspace and solution after 4 h and 20 h of incubation of Mc. capsulatus (Bath) in selenite-amended medium using sorptive extraction in conjunction TD-GC-MS analysis

Location of detection	Incubation time (h)	Detection of the following species^*a*^:										
		Methyl selenol	DMSeS	DMDSe	Bis(methylseleno) methane	Dimethyl selenosulfide	Dimethyl diselenenyl sulfide	Triselenothone/dimethyltriselenide	Benzothiazole	Diethyl sulfoxide	Propanesulfonyl	Dodecanethiol
Solution (selenite amended)	4	+	+	+	+	−	+	−	+	−	−	−
	20	+	+	+	+	+	+	+	+	+	−	−
Headspace (selenite amended)	4	+	+	+	−	−	−	−	+	−	−	−
	20	+	+	+	−	−	−	+	+	−	−	−
Control^*b*^	20	−	−	−	−	−	−	−	+	−	+	+

a The symbols denote detected (+) and undetected (−).

b The control is the same medium with no added selenite.

## DISCUSSION

The results from the previous kinetic experiments showed that there were increases in particle sizes with incubation time (25), leading us to hypothesize that much of the structure of the particles was formed in the extracellular space. If data support this hypothesis, then the key reactions resulting in the increase in the particle sizes are essentially abiotic in nature, though they require electrons ultimately derived from methane. Consequently, the clues to the structural formation of the particles must lie in the nature and the identity of the compounds that are concomitantly present in the solution and the headspace of the nascent particles. It was therefore essential to sample for selenium- and sulfur-containing compounds in both the headspace and the solution, followed by their analyses and identification.

The FTIR spectra of the SeNPs produced by Mc. capsulatus, samples of the biomass of the strain (control), as well as the Chem-SeNPs were recorded in order to identify the functional groups capping the synthesized SeNPs. These results were consistent with the particles being coated with cell-derived proteins and other biomolecules and are in line with their TEM images showing a thin layer over the particles, in addition to strong carboxylate bands, which may stabilize the SeNP structure and morphology.

The XPS results show two Se-containing species and other organic constituents. In assigning the Se bands, it is essential to link the structure of the particles to the selenium-containing species that have been identified in the solution (see the discussion of the TD-GC-MS results below). Since data for the exact compounds are not available, structures that may be similar to these in the particles have been selected. The major band at 55.16 eV has been assigned to the compound (CH_3_)_2_NC(Se)SeC(Se)N(CH_3_)_2_ (53). This is a reasonable fit to the results obtained in this study not only because of the presence of selenium but also because of the content of the methyl groups and nitrogen. The nitrogen could account for the single band at 400.10 eV usually assigned to amine nitrogen. The band at 56.02 eV has been assigned to [—CSeC(CH_3_)C(CH_3_)Se—]_2_ (54). Both of these assignments are an indication of the presence of a long chain of selenium-containing methylated species. Missing from both spectra is oxygen, which is presumably present as C=O and either C—O—C or C—O—H at 531.58 and 532.84 eV, respectively. However, the presence of Se—C was not detected in the spectrum, thus indicating that the amount of carbon directly bound to selenium was low. Furthermore, because of the high concentration of selenium in relation to sulfur, the signal for the latter could not be resolved and identified.

Whereas both FTIR and XPS provided information on the forms in which the elements are present on the particle surface, Raman spectroscopy enabled the identification of the basic structural makeup of the particles. The intensity of the band at 251.5 cm^−1^ is indicative of the predominance of the Se-Se bonds in the structure of the particles. It is probable that S is integral to the mixed particle structure Se_8 − *x*_S*_x_* and not present as an S_8_ impurity in the particles. The low available sulfur content in the medium makes the formation of an S—S homonuclear bond in the mixed particle structure highly unlikely (51).

We have previously reported that methyl selenol is produced and detected in the headspace only when selenite is in the starting medium (25). Methylated selenium-containing volatiles are known to be produced during the transformation of selenium-containing species by other types of bacteria (reviewed in reference 5), but the presence of methyl selenol has not been reported and may be unique to methanotrophs. Besides the presence of methyl selenol in the headspace, methyl selenoacetate was also detected. However, in the present study, methyl selenoacetate was not detected when the headspace sorptive extraction probes were deployed for sample collection. No methyl selenol was detected when either the harvested or the chemically synthesized nanoparticles were added to the medium in the absence of selenite. Therefore, we propose that methyl selenol may be the precursor of all of the methylated selenium species as well as the selenium-containing nanoparticles. If this is the case, the first step in the biotransformation of selenite by Mc. capsulatus would involve the reduction and methylation of selenite to methyl selenol, followed by the formation of the other selenium- and sulfur-containing species. Indeed, the formation of some of the latter species requires the presence of the nascent selenium particles.

**FIG 4:**
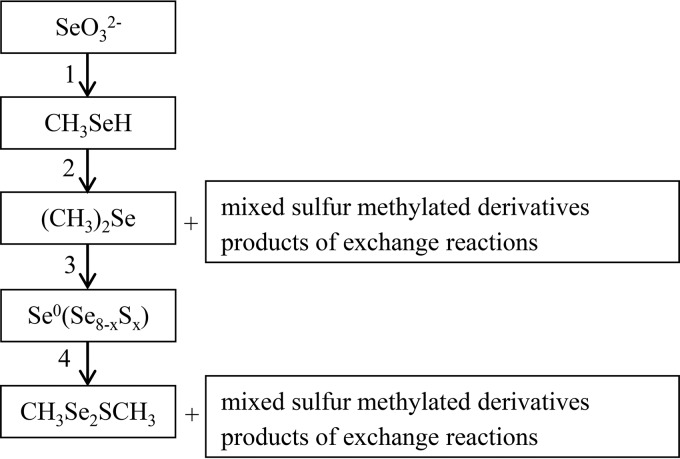
Schematic diagram showing the reduction of selenite to methyl selenol with the subsequent formation of other selenium-containing species. The numbers donate the following: 1, reduction and methylation; 2, reduction and methylation; 3, polymerization; 4, exchange reactions.

**FIG 5:**
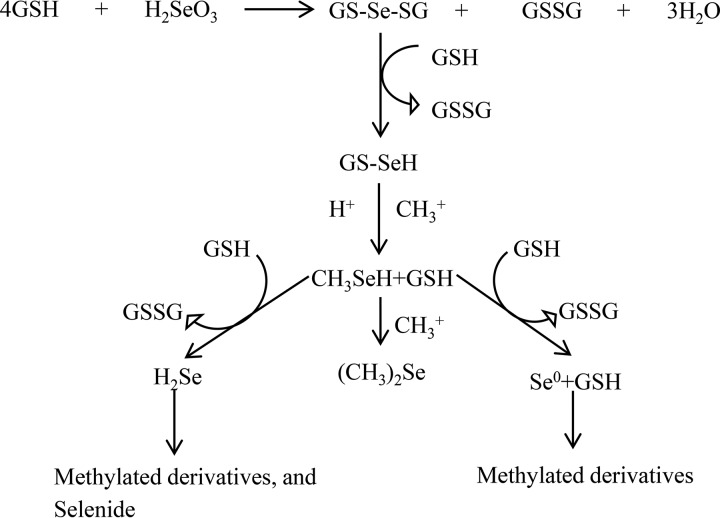
Schematic diagram showing the formation of methyl selenol, selenium particles, and methylated derivatives. Abbreviations: GSH, glutathione; GS-Se-SG, selenodiglutathione; GSSG, diglutathione; GS-SeH, selenoglutathione.

Based on these observations, a possible pathway for the formation of the particles and the other products of biotransformation of selenite can be outlined, as shown in Fig. 4. A series of reactions proposed by Ganther (55, 56) and outlined by Xu and Barton (22) implicates glutathione (GSH) and GSH reductase in the production of elemental selenium. This is possible in Mc. capsulatus, in which glutathione is known (57), though it would not account for possible differences in the pathway from that in other microorganisms in which GSH and GSH reductase are widespread. The proposed steps leading up to the formation of methyl selenol involving GSH are shown in Fig. 5. While the transformation of selenite by whole cells in culture requires the presence of the growth substrate methane, previous work showed that selenite is transformed by concentrated cell fractions (cell walls, membranes, and the soluble fraction) to the red particulate material in the absence of methane. The fact that the greatest activity was found in the cell wall-enriched fraction (25) is consistent with the extracellular growth of the particles.

The presence of the sulfur-containing benzothiazole was detected whether or not the culture had been amended with selenite. The sulfur-containing volatile species dodecanethiol and propanesulfonyl were detected only in the control cultures without selenite (Table 2). Such sulfur-containing species may be the source of sulfur in the structure of the selenium-rich particles. Evidence of the formation of the longer chains of the selenium-containing species can be seen in the nature of the compounds that were found in the solution after hours of incubation; first, after 4 h, dimethyl diselenenyl sulfide was detected, and subsequently, dimethylselenodisulfide, dimethyltriselenide, and bis(methylseleno) methane were detected after 20 h. More complex mixed Se and S compounds are formed in the medium with time. The formation of the mixed chalcogenides of Se and S is hardly surprising since S may be available from the reduction of sulfate in the growth medium. Genes for the assimilatory uptake and reduction of sulfate are present within the Mc. capsulatus genome (58), and the species grows using sulfate as the sole sulfur source. A key question, therefore, is how the solution chemistry relates to the observed structural features of the Se particulates.

It is likely that these longer chains polymerize to form Se*_x_* or Se_8 − *x*_S*_x_* linear or cyclic structures (32). Indeed, all chalcogen elements have the tendency to form cyclic allotropes (59). The dominant allotrope depends on the experimental conditions. Examples of exchange reactions that may occur based on the presence of the detected selenium- and sulfur-containing species are shown in the following equations:

(1) $$\text{-Se-Se- + 2-Se-Se-Se-}\to {\text{Se}}_{8}$$

(2) $$\text{-Se-S- + 2-Se-Se-Se-}\to {\text{ Se}}_{7}\text{S}$$

(3) $$\text{-Se-Se- + 2-Se-Se-S-}\to {\text{Se}}_{6}{\text{S}}_{2}$$

As can be seen, a variety of nanocomposites can be formed. It is noteworthy that these structures are methylated, providing an organic coating associated with the nanoparticles (60). Similar exchange reactions have been shown to occur when mixed Se and S complexes are present in the same solution (32). Indeed, these reactions are known to occur in amorphous selenium semiconductors (61). The Raman spectroscopy results indicate that these are not open-chain clusters but are cyclic structures in which the Se_8_ structure is dominant and in which there is the probable presence of small amounts of Se-S bonds. The source of sulfur must be from the reduction of sulfate used in the growth medium since this is the only sulfur added to the system. If it is desired to reduce the sulfur content of the particles, it may be possible to achieve this by reducing the amount of sulfate in the culture medium so that sulfur becomes growth limiting. Since it may be that the cells can produce the nanoparticles after they have ceased to grow, this may allow the selenite to be added to the medium after the sulfate is used up, so that less sulfur would be available for incorporation into the particles.

In this study, we demonstrate for the first time that in the reduction of selenite by Mc. capsulatus (Bath), a methane-oxidizing bacterium, methyl selenol is the likely precursor for the formation of methylated selenium-containing and mixed chalcogenide species. This reaction may be biotechnologically useful for the bioremediation of selenite and the production of selenium-sulfur nanoparticles for electronics and other applications, using cheaply available methane as the feedstock. If this reaction is widespread among methanotrophs, it may also have significance in selenium transformations in the environment. To date, it has been shown that four isolated strains, including methanotrophs of the gammaproteobacteria (Mc. capsulatus Bath, Methylomicrobium buryatense, and Marinospirillum alkaliphilum) and alphaproteobacteria (Ms. trichosporium OB3b), perform this reaction.

Subsequent exchange reactions between the species result in the formation of the amorphous allotropic form of selenium, cyclic Se_8_, with sulfur in its structure. The nature of the molecular mediators in the reduction of selenite supplying sulfur that is integral to the structure of the nanoparticles and supplying the methyl groups found in the volatile selenium-containing products remains to be identified.

## MATERIALS AND METHODS

### Bacterial strains and growth conditions.

The methanotrophic bacterium Methylococcus capsulatus (Bath) (NCIBM 11132) was grown and propagated aerobically using methane as the carbon and energy source as previously described (25). For these experiments, the initial selenite concentration used was 20 mg liter^−1^.

### Detection of solution and volatile selenium-containing species.

Solution and volatile selenium-containing species were sampled by immersive sorptive extraction using sampling probes (HiSorb probe; Markes International Limited, UK) from either the solution or the headspace. Extension screw-on arms were fabricated for each probe so that they could be inserted through the Suba-Seals used to seal the necks of the culture flasks. To ensure that the probes and the tubes were contamination free, before use, the probes and tubes were preconditioned with helium at a flow rate of 90 ml min^−1^ using the following temperature program: 15 min at 100°C, 15 min at 200°C, 15 min at 300°C, and 15 min at 335°C.

The preconditioned probes were inserted into either the liquid or the headspace of the Mc. capsulatus (Bath) culture medium through the Suba-Seals. The probes were removed from the Suba-Seals after different incubation times (4 and 20 h), rinsed with high-performance liquid chromatography-grade water, dried with lint-free tissue, and then placed into the thermal desorption tubes (Markes International Limited, UK).

Sample analyses were performed on a combined thermal desorption GC-MS system. The volatiles were desorbed at 250°C and concentrated on a thermal desorber (Unity; Markes International Limited) in a −10°C cold trap for 5 min (helium flow rate, 50 ml min^−1^) and then were transferred onto the GC-MS system (a model 7890A GC with a model 5975C MS; Agilent Technologies) equipped with a capillary column (an Agilent J&W HP-5MS GC column; 30 m, 0.25 mm, 0.25 μm). Helium was used as the carrier gas at a flow rate of 1 ml min^−1^ and an injector temperature of 250°C, and the chromatogram was obtained using the following temperature program: 35°C for 1 min, 10°C min^−1^ to 250°C, and then a hold at 250°C for 1 min. The National Institute of Standards and Technology (NIST) MS search program (version 2011) was used to identify the compounds based on their MS spectra.

### Extraction of selenium nanoparticles produced by Mc. capsulatus (Bath).

Freshly grown cultures (optical density at 600 nm [OD_600_], 0.5 to 0.8) were supplemented with 20 mg liter^−1^ SeO_3_^2−^, and incubation was continued at 45°C with shaking in the presence of methane. After 48 h, the development of the reddish color had occurred and the cultures were pelleted by centrifugation (at 12,500 × *g* for 10 min). SeNPs were extracted by a modification of the method published by Sonkusre et al. (62), as follows. The resultant pellet was washed and resuspended in 10 ml of sterile water, followed by the addition of lysozyme to give a final concentration of 500 μg ml^−1^, and the tube was incubated at 37°C for 3 h. The suspension was passed through a French pressure cell (1,500 lb/in^2^, 4°C). The resultant slurry, containing both cell debris and NPs, was washed four times by centrifugation at 15,000 × *g* for 10 min with 1.5 M Tris-HCl (pH 8.3) containing 1% sodium dodecyl sulfate (SDS). The resultant pellet, containing SeNPs and the insoluble cell wall fraction, was washed and resuspended in 4 ml sterile water in a 15-ml Falcon tube, and 2 ml of 1-octanol was added. The solution was mixed vigorously on a vortex mixture for 5 min and centrifuged at 2,000 × *g* for 5 min at 4°C. The tubes were then kept undisturbed at 4°C for 24 h. The upper phase and interface, containing the insoluble cell fraction, were removed, and the bottom water phase, containing SeNPs, was transferred to a clean 15-ml centrifuge tube. This was washed sequentially with chloroform, absolute ethanol, 70% ethanol, and water by centrifugation at 16,000 × *g*. The collected NPs were resuspended in water and stored at 4°C.

### TEM and EDX spectrometry/HAADF-STEM analysis.

Samples of selenite-amended culture were treated and analyzed as previously described (25). The samples were examined in an FEI Tecnai F20 field emission gun (FEG) transmission electron microscope operating at 200 kV and fitted with a Gatan Orius SC600A charge-coupled-device (CCD) camera, an Oxford Instruments XMax SDD energy-dispersive X-ray (EDX) detector, and a high-angle annular dark-field (HAADF) scanning transmission electron microscopy (STEM) detector.

For thin-section analysis, after the ethanol dehydration steps, the cells were embedded in EMbed 812 epoxy resin and cut into thin sections (90 nm, using a diamond knife on a Reichert Ultracut S ultramicrotome). The sections were supported on copper grids and coated with carbon. TEM specimen holders were cleaned by plasma prior to TEM analysis to minimize contamination. Samples were examined with a high-resolution Philips CM 200 transmission electron microscope at an acceleration voltage of 200 kV under standard operating conditions with the liquid nitrogen anticontaminator in place.

### X-ray absorption spectroscopy.

The conditions for the X-ray absorption spectroscopy measurements were as described previously (25).

### XPS analysis.

Harvested SeNP samples were deposited on a silicon wafer and left to dehydrate in the load lock of the X-ray photoelectron spectroscopy (XPS) instrument overnight. The analyses were carried out using a Kratos Axis Ultra DLD instrument with a monochromated aluminum source. Survey scans were collected at binding energies of between 1,200 and 0 eV at a 160-eV pass energy and 1-eV intervals. High-resolution C 1s, N 1s, O 1s, Se 3d, and S 2p spectra were collected over an appropriate energy range at a 20-eV pass energy and 0.1-eV intervals. The analysis area was 700 μm by 300 μm. Two areas were analyzed for each sample, with the data being collected in duplicate. Charge neutralization was used with the intention of preventing excessive charging of the samples during analysis. The data collected were calibrated in intensity using a transmission function characteristic of the instrument (determined using software from NPL) to make the values instrument independent. The data were then quantified using theoretical Schofield relative sensitivity factors. The data were calibrated for binding energy using the main carbon peak C 1s at 285.0 eV as the reference peak and correcting all data for each sample analysis accordingly.

### Raman spectroscopy analysis of SeNPs.

Aliquots of 2 μl of SeNPs suspended in water were transferred onto a calcium fluoride (CaF_2_) slide and air dried prior to Raman spectroscopy analysis. Raman spectra were obtained using a Horiba LabRam HR spectrometer and a modified Horiba LabRam HR spectrometer (Wellsens Biotech Ltd., China). Three factors have been modified in this new Raman system to improve Raman spectral quality. These comprise shortening the Raman light path, employing a low-noise and sensitive electron-multiplying CCD (EMCCD) for Raman signal detection, and increasing the incident laser power. The old and new modified systems are identical except for these three factors. The Raman signals were collected by a Newton EMCCD (model DU970N-BV; Andor, UK), utilizing a 1,600-by-200 array of 16-μm pixels with thermoelectric cooling down to −70°C for a negligible dark current. A 532-nm neodymium-doped yttrium aluminum garnet (Nd:YAG) laser (Ventus, Laser Quantum Ltd., UK) was used as the light source for measurement of the Raman spectra. A 100× magnifying dry objective (numerical aperture = 0.90; Olympus, UK) was used for sample observation and Raman signal acquisition. A 600-line/mm grating was used for the measurements, resulting in a spectral resolution of ∼1 cm^−1^ with 1,581 data points. The laser power on the sample was measured by a laser power meter (Coherent Ltd.). The Raman spectra were processed by background subtraction (the background was determined using the spectra from a cell-free region on the same slide) and normalization using Labspec5 software (Horiba Jobin Yvon Ltd., UK).

### FTIR spectroscopy measurements of SeNPs.

In order to determine the functional groups present on the SeNPs, the Fourier transformation infrared (FTIR) spectra of the SeNPs were recorded on a PerkinElmer Spectrum 100 FTIR spectrometer equipped with an attenuated total reflectance (ATR) attachment. The spectra were recorded from 4,000 to 650 cm^−1^, and 4 scans were averaged at a resolution of 4 cm^−1^. Extracted SeNPs were freeze-dried overnight and analyzed without further treatment. For comparison, the FTIR spectra of samples of bacterial cells (as a control) and chemically synthesized SeNPs (Chem-SeNPs) were also recorded. For the controls, freshly grown cultures (OD_600_, ∼0.7) of Mc. capsulatus (Bath) were centrifuged at 11,000 × *g* for 10 min to obtain the cell pellets. The pellets were washed twice with phosphate-buffered saline (sodium chloride, 150 mM sodium phosphate, 150 mM), pH 7.2, and then freeze-dried overnight. The synthesis of Chem-SeNPs was done according to a previously described procedure of (62), as follows: 1.0 ml of a 50 mM l-cysteine (Sigma-Aldrich, Dorset, UK) solution was added dropwise into 1.0 ml of 0.1 M Na_2_SeO_3_. The mixed solution was then stirred for 30 min at room temperature. The Chem-SeNPs were pelleted by centrifugation (at 15,000 × *g* for 10 min) and then freeze-dried overnight.

## Supplementary Material

Supplemental file 1
