# Supplementary material for: Methyl Selenol as a Precursor in Selenite Reduction to Se/S Species by Methane-Oxidizing Bacteria
Source: Appl Environ Microbiol. 2019 Oct 30;85(22):e01379-19. doi: 10.1128/AEM.01379-19 (PMC6821961; doi:10.1128/AEM.01379-19)

Supplemental Figure 1. Wide scan X-ray photoelectron spectra of the SeNPs produced by *Mc. capsulatus* (a) and high resolution spectra for Se 3d, C 1s, O 1s and N 1s are shown in b, c, d and e, respectively. The spectra are representatives of 2 runs of the experiments. The regions of the wide scan spectrum that are shown in greater detail in the high resolution spectra (b-e) are indicated in part (a). Experimental data are shown by the red lines. Deconvoluted data for the different components of spectra in parts b-e are shown in different

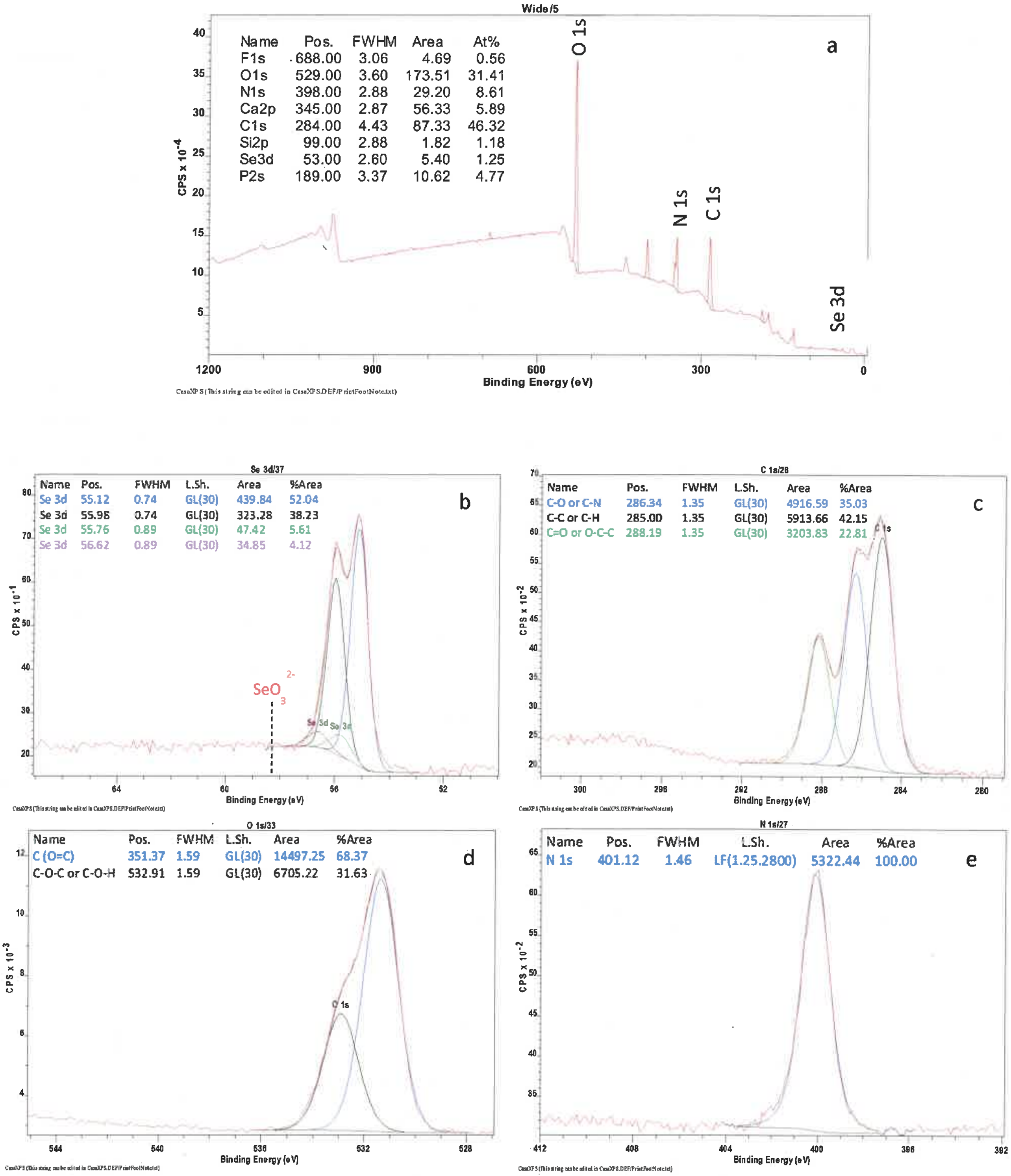

colours as indicated in each panel. Binding energy of electrons is shown in electron volts (1 eV = 1.60×10^{-19} J).

Supplemental Figure 2

The Raman spectra of purified Se nanospheres from *Mc. capsulatus* after 24h incubation with deconvoluted data for the different components of spectra.

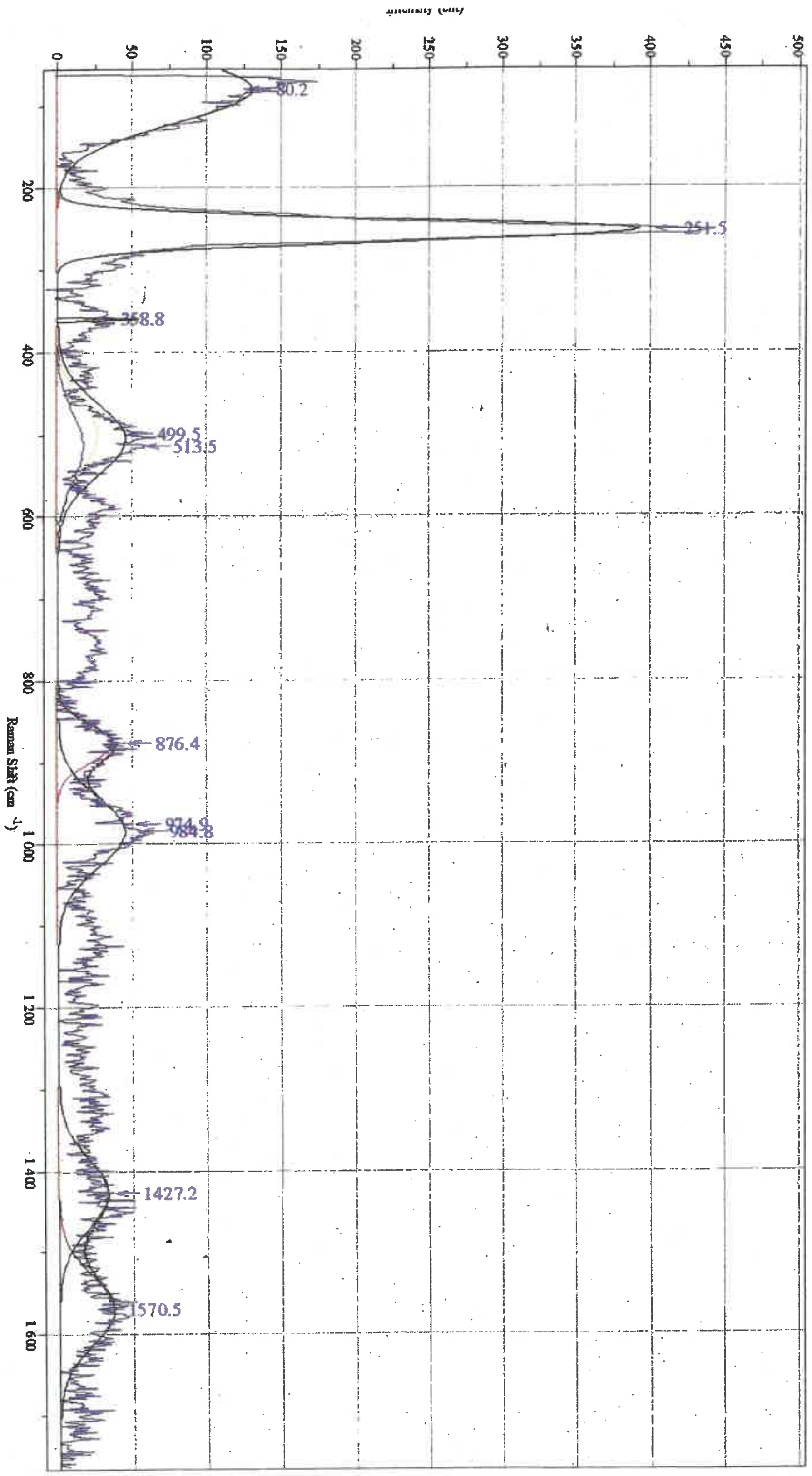

Supplemental Figure 3 GC mass chromatograms of the liquid phase and headspace of the *Mc. capsulatus* (Bath) cultures amended with selenite (20 mg L^{-1}) at 4 h and 20 h. The chromatograms were obtained by selecting the 80 m/z ion, and peak identification was achieved using the GC-MS library.

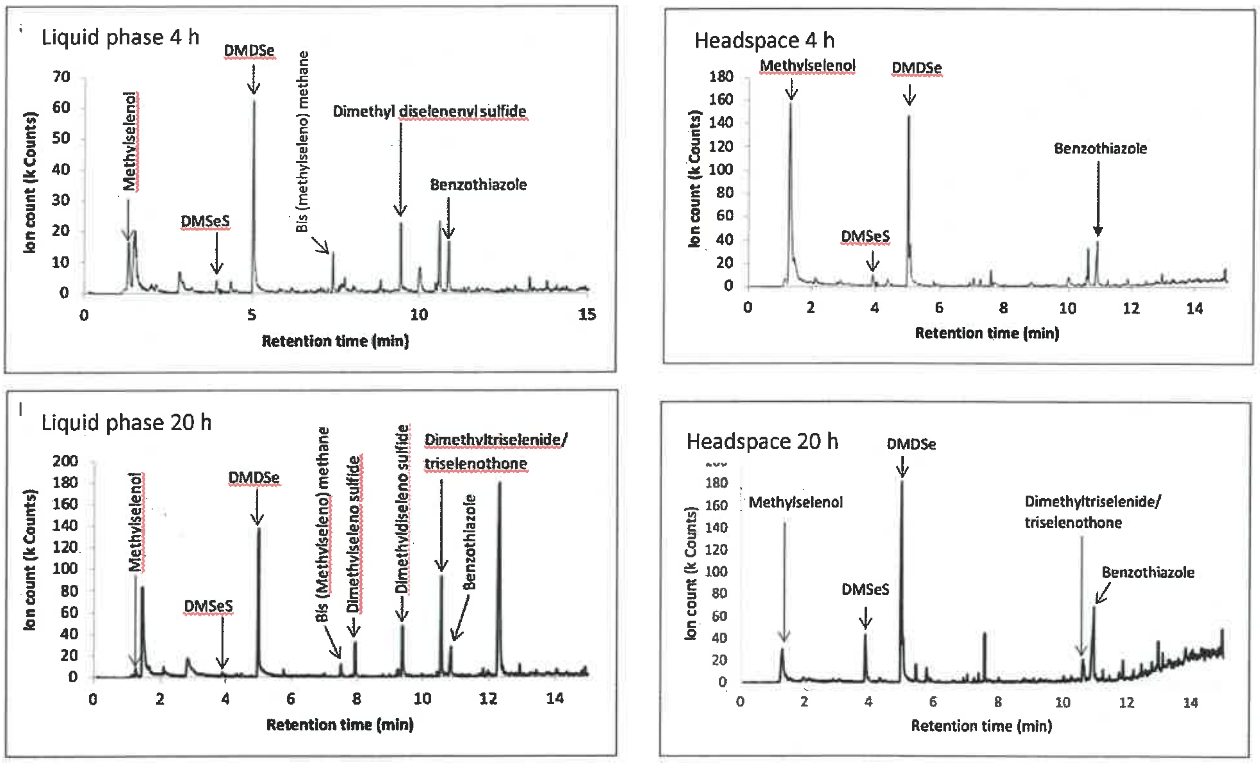

Supplement: Supplemental file 1 [file AEM.01379-19-s0001.pdf]
